# Cross-sectional evaluation of FGD-avid polyostotic fibrous dysplasia: MRI, CT and PET/MRI findings

**DOI:** 10.1186/s41824-022-00139-0

**Published:** 2022-10-03

**Authors:** Chiara Pozzessere, Francesco Cicone, Paolo Barberio, Annalisa Papa, Giuseppe Coppolino, Roberto Biagini, Giuseppe Lucio Cascini

**Affiliations:** 1 Radiology Unit, AUSL Toscana Centro San Giuseppe Hospital, Empoli, Italy; 2 Nuclear Medicine Unit, University Hospital “Mater Domini”, Catanzaro, Italy; 3 Department of Experimental and Clinical Medicine, “Magna Graecia” University of Catanzaro, Catanzaro, Italy; 4 PET/MR Unit, Neuroscience Research Centre, “Magna Graecia” University of Catanzaro, Catanzaro, Italy; 5 Nephrology and Dialysis Unit, “Magna Graecia” University of Catanzaro, Catanzaro, Italy; 6 Oncological Orthopaedics Department, IRCCS Regina Elena National Cancer Institute, Rome, Italy

**Keywords:** Positron emission tomography, FDG, Magnetic resonance imaging, Polyostotic fibrous dysplasia, Differential diagnosis

## Abstract

A 42-year-old male with left hip pain was diagnosed of several right femoral and tibial bone tumours. All lesions were osteolytic with sclerotic margins. The symptomatic lesion in the proximal femur also showed bone expansion and focal cortical thinning. Whole-body [^18^F]-fluorodeoxyglucose (FDG) PET/CT and segmental PET/MRI of the left hip and femur were performed for metabolic characterization of the lesions and for biopsy guidance. The lesions showed a heterogenous degree of FDG uptake corresponding to different metabolic stages of the disease. A biopsy of the tumour portion showing the highest FDG uptake revealed a fibrous dysplasia (FD). In conclusion, although generally affecting paediatric and adolescent subjects, polyostotic FD may be detected in the adulthood. Despite the benign nature of the disease, increased glucose metabolism can be seen in some lesions. Hybrid imaging combining morphological and functional information may help guide biopsy and better define the treatment strategy.

## Case presentation

A 42-year-old male patient was referred for a magnetic resonance (MRI) of the left hip because of pain with no signs of movement limitations. A lesion was discovered along the lesser trochanter and proximal diaphysis of the left femur. The lesion was characterized by bone expansion, thinned borders, low signal intensity on T1-weighted (T1w) sequences, heterogeneous signal on T2-weighted (T2w) sequences, with solid, slightly hypointense components and hyperintense areas. Moreover, endosteal scalloping and focal cortical thinning were seen, with suspected cortical interruption of the posterior border of the lesser trochanter. Based on these findings, a definitive radiological diagnosis could not be established.

A computed tomography (CT) was performed to better characterize the nature and the extension of the lesion. It appeared as a well-defined osteolytic lesion, large and centrally located, with ground-glass matrix, bone expansion, endosteal scalloping and focal cortical thinning particularly in the posterior edge of the lesser trochanter. No periosteal reaction was seen. Two additional osteolytic lesions were detected in the left femur, one in the distal diaphysis and one in the distal metaphysis, respectively, and another one in the proximal metaphysis of the left tibia. These lesions were eccentrically located, osteolytic with well-defined sclerotic and sharp borders, with no periosteal reaction. The multifocal bone involvement was evocative of polyostotic bone tumours.

**Fig. 1:**
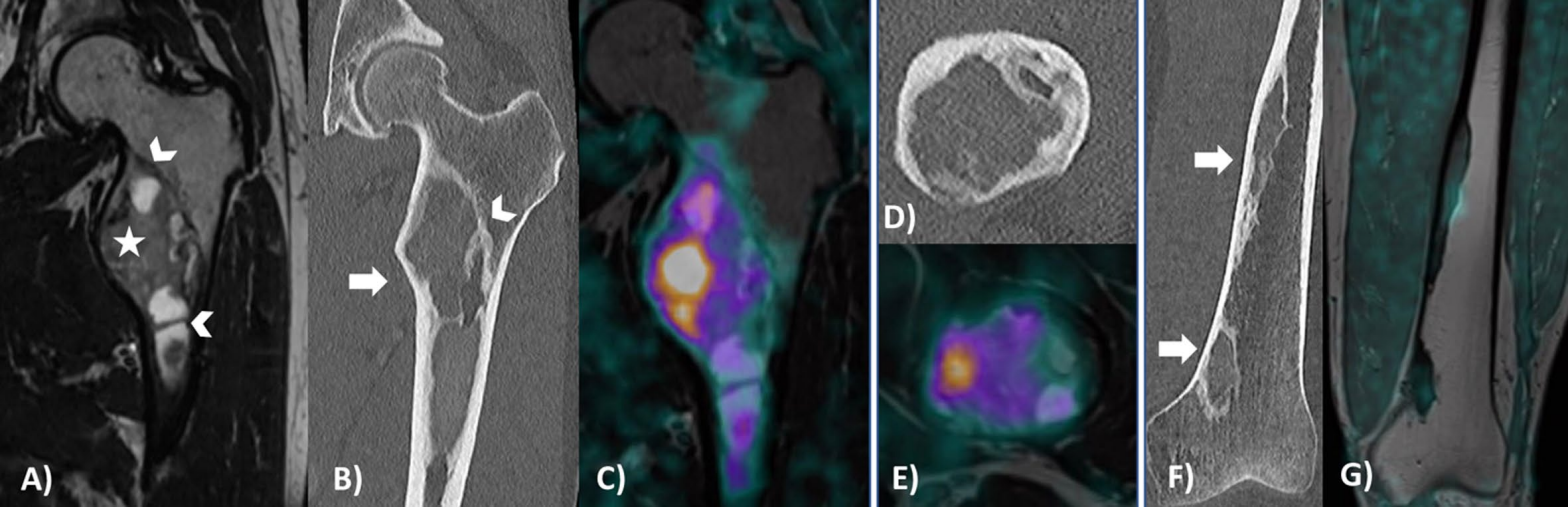
Cross-sectional imaging of left femoral bone lesions. T2-weighted, coronal MR (**A**), CT (**B**), and PET/MR (**C**) images showing a large lesion of the lesser trochanter and proximal diaphysis of the left femur, characterized by increased FDG metabolism corresponding to the viable portion of the tumour. Axial CT suggests cortical interruption of the posterior border of the lesser trochanter (**D**), corresponding to metabolically inactive tissue, as shown by PET/MRI (**E**). Coronal CT (**F**) showing two additional osteolytic lesions in the distal diaphysis and in the distal metaphysis of the left femur, respectively, with well-defined sclerotic borders and no periosteal reaction. T1-weighted PET/MRI fusion sequence (**G**) shows no significant FDG uptake corresponding to these additional lesions. FDG images were adjusted to the same intensity colour scale

The patient was subsequently referred for a whole-body [^18^F]-fluorodeoxyglucose (FDG) PET/CT (Discovery ST 8 slice -2D, GE Healthcare, Boston, USA), to evaluate the metabolic activity of the lesions and to exclude the involvement of other bone segments. Following the PET/CT session, and using the same FDG injection, a FDG PET/MRI (Siemens Biograph mMR, Siemens Healthcare, Erlangen, Germany) of the left hip and leg was acquired for biopsy planning. The known lesions showed heterogeneous FDG uptake due to different metabolic stages of the disease. High metabolic activity (SUVmax = 3.7) was seen in the solid component of the upper lesion, whereas there was no increased uptake corresponding to the area of cortical interruption in the posterior border of the lesser trochanter. The additional lesions identified showed lower FDG uptake (SUVmax = 1.3 -1.4), only mildly increased over the background signal (Fig. 1). A surgical biopsy of the proximal femoral lesion was performed at the site of maximum FGD uptake, showing pathological findings consistent with fibrous dysplasia (FD). The diagnosis was confirmed after a second histological look. At the time of writing, the patient has been followed conservatively for more than one year.

## Differential diagnosis

Radiological modalities such as radiography and CT play a major role in the work-up of bone tumours. Five features should be evaluated for a correct differential diagnosis of osteolytic lesions: (i) patient’s age; (ii) symptoms; (iii) location; (iv) biological activity; and (v) matrix of the lesion (Miller 2008). Age is a pivotal characteristic in the differential diagnosis. In fact, while malignant tumours, particularly metastasis and multiple myeloma (MM), represent the most common cause of bone lesions in adults, bone tumours in young adults and children are most frequently benign. Tumour location is important as some bone tumours have a typical tropism for certain bones, such as the proximal femur and the knee. Similarly, location within a bone—epiphysis, metaphysis and diaphysis—is an important landmark, although many tumours, such as FD, may involve both metaphysis and diaphysis. Borders and zone of transition should be considered to assess lesion biological activity. Geographic lysis with well-defined borders and narrow zone of transition defines a slow-growth lesion, typical sign of benignity. Sclerotic reaction of the surrounding bone represents the bone response to the tumour, being absent or insufficient when the growth of the tumour is aggressive. The pattern of cortical involvement (thinning, interruption, disruption or infiltration) has relevance in determining the risk of fracture and tumour biological activity. Regular destruction and endosteal scalloping can be found in benign lesions. Matrix mineralization depends on the tissue of origin, whether fibrous with ground-glass appearance, osteoid with cumulus cloud, or chondroid with pop-corn calcifications (Miller 2008).

In the present case, several bone lesions were found. The tumours were unilateral, located in the metaphysis and the diaphysis of the femur and tibia. The largest lesion, located in the proximal femur, was symptomatic, showing ground-glass matrix, bone expansion, endosteal scalloping and focal cortical destruction. In contrast, the other lesions were well-defined, osteolytic with sclerotic borders, ground-glass matrix and no periosteal reaction. Hybrid imaging revealed increased FDG uptake corresponding to the solid component seen at MRI, confirming high biological activity at that level. A malignant degeneration could not be excluded by imaging. Conversely, the portion of the lesion showing bone cortical interruption was not characterized by significantly increased glucose metabolism.

The multifocality and unilaterality of the lesions are suspicious for polyostotic tumours. Among the polyostotic lesions, FD, non-ossifying fibromas (NOF), multiple enchondromas, bone infarcts, and infections could be evocated (Miller 2008; Nascimento et al. 2014; Costelloe et al. 2014). Infectious tumour-like lesions were excluded by clinical and laboratory findings. Malignant bone tumours such as metastases, lymphoma and MM were unlikely due to the low-aggressive CT features. The patient was in his 4th decade, which is a borderline age for bone tumours. It is quite late for asymptomatic polyostotic disease which, however, could not be excluded “a priori” (Miller 2008). Imaging findings of the more distal lesions were similar to those of multiple NOF, enchondromas and bone infarction. NOF are non-neoplastic fibrous tumours which may be incidentally detected in children and adolescent and usually disappear before the age of thirty. Multiple NOF are associated with neurofibromatosis type 1 (NF1) and Jaffe–Campanacci syndrome. On imaging, they appear as multiloculated lytic lesions with sclerotic borders, with no periosteal reaction, generally eccentrically located in the metaphysis and in the diaphysis of the long bones.^1^ At MRI, they are generally hyperintense on long TR sequences, although they can change over time becoming hypointense on T2w in the late phase (Nascimento et al. 2014). Although NOF are metabolically inactive, FDG uptake may be seen in case of fracture or in the reabsorption phase (Costelloe et al. 2014). Enchondromatosis is benign cartilage tumours affecting children and young adults. Generally asymptomatic, they turn painful in case of fracture or — rarely— malignant degeneration in chondrosarcoma. Multiple enchondromas are associated with Maffucci syndrome and Ollier disease. The lesions appear as lytic lesions with narrow zone of transition and sharply borders, generally affecting the metaphysis and the diaphysis of the long bones in centric or eccentric location (Miller 2008). Chondroid calcification, also called “rings and arcs sign”, and endosteal scalloping may be seen. Phalanx, humerus, radio, ulna femurs and tibia are frequently involved. On MRI, T1w and T2w hypointense foci within the lesion can be seen, representing the chondroid matrix (Nascimento et al. 2014). These lesions may show FDG uptake (Costelloe et al. 2014). In our case, the absence of a chondroid matrix made enchondromatosis unlikely. Bone infarctions may be incidentally detected in the metaphysis and appear as lytic medullary lesions surrounded by sclerotic serpiginous borders (Miller 2008). MRI is useful in the diagnosis since they typically present a hypointense peripheral serpiginous border on T1w and T2w, an inner ring of granulation hypointense on T1w and hyperintense in T2w, while maintaining the central signal of the marrow in both T1w and T2w (Nascimento et al. 2014). In the present case, MRI was useful to reject this hypothesis.

## Discussion

Both malignant and benign bone tumours, as well as tumour-like lesions, can show increased FDG uptake depending on their biological activity. These include bone cysts, geodes, haemangiomas, bone infarcts, bone island, enchondromas, FD, osteochondroma, Paget’s disease and bone infections (Elangovan and Sebro 2019). Although increased glucose metabolism may be suspicious for malignancy, a correct differential diagnosis requires the pattern of FDG uptake to be always considered in the specific clinical context together with the radiological appearance of the lesion. FDG PET imaging can also be successfully used for biopsy guidance of bone tumours (Wu et al. 2019). PET allows guiding the needle within the hypermetabolic area of the lesions, which represents the viable, cellular-rich portion of the tumour. Compared to CT-guided biopsy, PET/CT-guided biopsy results in higher rate of diagnostic specimens (Wu et al. 2019). PET/MRI, a low-dose hybrid imaging modality, provides tissue characterization along with metabolic information, allowing the identification of different lesion components, such as cystic, necrotic, haemorrhagic or viable components. Bone and soft tissue disease may represent one of the most relevant fields of application of PET/MRI (Kogan et al. 2018; Eiber et al. 2014). In the present case, a PET/MRI segmental acquisition was performed straight after whole-body PET/CT. Therefore, an optimal depiction of the most active tumour portion was obtained by adding no further radiation dose to the patient. FD is a congenital benign skeletal disorder characterized by immature bone and fibrous tissue replacement devoid of hematopoietic marrow, caused by the sporadic mutation of a protein involved in the osteoblastic differentiation, called GNAS (Bianco et al. 2000). FD may be solitary (monostotic) or multiple (polyostotic) tumour-like lesions. Monostotic form accounts for almost 80% of cases, and lesions are generally asymptomatic and incidentally discovered before the age of 30. However, the replacement of normal bone may be clinically evident due to important bone expansion and/or compression and dislocation of the adjacent structures or may cause fractures. Malignant dedifferentiation is a rare event, described in previously irradiated patients. Polyostotic disease is usually symptomatic and diagnosed during the childhood, and this form may be sporadic or associated with endocrinopathies or systemic disorders such as Mc Cune Albright syndrome and Mazabraud syndrome. FD lesions are dynamic, changing their features over time till adulthood when they become inactive and regress. FD typically arise in the diaphysis or in the metadiaphysis of long bones. Rib, proximal femur, tibia, craniofacial bones and humerus are the most frequent locations of monostotic FD. Polyostotic lesions are often unilateral; femur, tibia, pelvis, foot and ribs are most frequently involved. In particular, femur and tibia are often simultaneously affected. On imaging, FD may appear as long well-defined lytic lesions with ground-glass matrix, sclerotic borders and a small zone of transition (Kushchayeva et al. 2018). Periosteal reaction is rare, whereas endosteal scalloping and focal cortical destruction are common. It may cause bone expansion and bone deformity or pathologic fracture. In the inactive phase, FD may result in sclerotic lesions. Beside the wide morphological variability secondary to its dynamism, FD may show heterogeneous FDG uptake resulting from its metabolic activity (Su et al. 2011). As demonstrated by both radiological and metabolic findings, in the present case, the lesions were at different evolutionary stages. The lesions located in the distal femur and in the proximal tibia were in the inactive phase, as confirmed by the absence of FDG uptake and by the well-defined sclerotic margins. In contrast, the lesion located in the proximal femur, which was painful, showed bone expansion as well as high FDG avidity as result of local biological activity, with potentially increased risk of pathologic fracture at that level.

## Conclusion

Radiograph and CT represent the modalities of choice for bone lesion assessment, and however, the interpretation of anatomical imaging may be challenging in some cases requiring further evaluation and biopsy to achieve a definitive diagnosis. Hybrid imaging combining anatomical and functional information, including PET/MRI, may be useful for biopsy guidance. Physicians reporting hybrid scans must be aware that, although generally affecting paediatric and adolescent subjects, both monostotic and polyostotic FD may be detected in the adulthood. FDG uptake can be seen in bone tumours, irrespectively of the malignant nature, depending on the biological activity of the lesion.

## Data Availability

Not applicable.

## Abbreviations

FDG: [^18^F]-fluorodeoxyglucose
CT: Computed tomography
PET/CT: Positron emission tomography–computed tomography
PET/MRI: Positron emission tomography–magnetic resonance imaging
SUVmax: Maximum standardized uptake value, normalized to patient’s body weight
FD: Fibrous dysplasia
MRI: Magnetic resonance imaging
MM: Multiple myeloma
NOF: Non-ossifying fibromas
NF1: Neurofibromatosis type 1
